# The Scavenging Effect of Myoglobin from Meat Extracts toward Peroxynitrite Studied with a Flow Injection System Based on Electrochemical Reduction over a Screen-Printed Carbon Electrode Modified with Cobalt Phthalocyanine: Quantification and Kinetics †

**DOI:** 10.3390/bios11070220

**Published:** 2021-07-02

**Authors:** Ioana Silvia Hosu, Diana Constantinescu-Aruxandei, Florin Oancea, Mihaela Doni

**Affiliations:** 1 Bioproducts Department, National Institute for Research & Development in Chemistry and Petrochemistry—ICECHIM, 202 Spl. Independentei, Bioproducts, Sector 6, 060021 Bucharest, Romania; diana.constantinescu@icechim.ro (D.C.-A.); florin.oancea@icechim.ro (F.O.); 2 Biotechnology and Bioanalysis Department, National Institute for Research & Development in Chemistry and Petrochemistry—ICECHIM, 202 Spl. Independentei, Sector 6, 060021 Bucharest, Romania

**Keywords:** electrocatalysis, peroxynitrite, flow injection analysis, meat extracts, myoglobin, cobalt phthalocyanine, electrochemical reduction, screen-printed carbon electrode, amperometric detection, decay kinetics

## Abstract

The scavenging activity of myoglobin toward peroxynitrite (PON) was studied in meat extracts, using a new developed electrochemical method (based on cobalt phthalocyanine-modified screen-printed carbon electrode, SPCE/CoPc) and calculating kinetic parameters of PON decay (such as half-time and apparent rate constants). As reactive oxygen/nitrogen species (ROS/RNS) affect the food quality, the consumers can be negatively influenced. The discoloration, rancidity, and flavor of meat are altered in the presence of these species, such as PON. Our new highly thermically stable, cost-effective, rapid, and simple electrocatalytical method was combined with a flow injection analysis system to achieve high sensitivity (10.843 nA µM^−1^) at a nanomolar level LoD (400 nM), within a linear range of 3–180 µM. The proposed biosensor was fully characterized using SEM, FTIR, Raman spectroscopy, Cyclic Voltammetry (CV), Differential Pulse Voltammetry (DPV), and Linear Sweep Voltammetry (LSV). These achievements were obtained due to the CoPc-mediated reduction of PON at very low potentials (around 0.1 V vs. Ag/AgCl pseudoreference). We also proposed a redox mechanism involving two electrons in the reduction of peroxynitrite to nitrite and studied some important interfering species (nitrite, nitrate, hydrogen peroxide, dopamine, ascorbic acid), which showed that our method is highly selective. These features make our work relevant, as it could be further applied to study the kinetics of important oxidative processes in vivo or in vitro, as PON is usually present in the nanomolar or micromolar range in physiological conditions, and our method is sensitive enough to be applied.

## 1. Introduction

**Figure 1:**
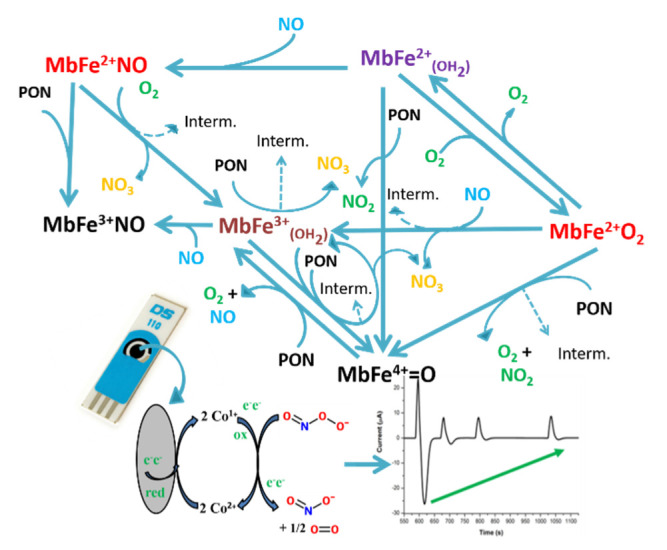
Graphical abstract. ferrylmyoglobin: MbFe^4+^ = O, oxymyoglobin: MbFe^2+^O_2_, deoxymyoglobin: MbFe^2+^(OH_2_), metmyoglobin: MbFe^3+^(OH_2_), nitrosylmetmyoglobin: MbFe^3+^NO, nitrosylmyoglobin: MbFe^2+^NO. This is a schematic representation of the chemical reactions of different forms of myoglobin with peroxynitrite and other interfering species/decomposition products. This scheme is not exhaustive and was inspired from data from different literature references [2,21,22,23,24,25,26].

For the food industry and for the consumers, it is very important to monitor the quality and freshness of raw meat. Different factors are a sign of meat alteration (e.g., discoloration, rancidity, alteration of flavor) [1,2,3]. One pathway of alteration is the scavenging activity of myoglobin toward nitro-oxidative species (such as peroxynitrite, PON). For example, the formation of metmyoglobin can alter the flavor due to lipid and protein oxidation [4]. The lack of metmyoglobin (MbFe^3+^OH_2_ or metMb) reducing enzymatic systems in meat after slaughter determines the irreversibility of the oxidation processes of myoglobin [5]. The color changes are a sign of these processes, and some possible oxidation pathways are described in Figure 1 [6]. The aspect of meat, by itself, has a great impact on consumers, and the impact on the food industry is huge. Adding nitrites to the raw meat helps keeping the pink color of the meat, as NO (nitric oxide) can bind to the iron ion in a similar way as oxygen molecule does. Nitrites and nitrates are also two of the decomposition compounds of peroxynitrite. Distinguishing between these species is important for meat quality.

The detection of peroxynitrite, being a short-living ROS in biological samples, is a big challenge that scientists still try to solve nowadays. Even if there are different methods of detection presented in the literature, most of them rely on indirect methods after the formation of secondary species. Forming different species, such as 3-nitrotyrosine, that can be detected by immunochemical or chromatographic techniques, or oxidizing different probes with peroxynitrite, further to be detected with fluorescent and chemiluminescent methods, are the usual methods [7]. The problem is that the selectivity toward peroxynitrie is not assured using these methods, as other ROS/RNS species could give the same response. Other usually used methods are high-performance liquid chromatography, UV-Vis absorbance spectroscopy, electron spin resonance, and electrochemistry [8]. These methods usually use antioxidants such as resveratrol, polyphenols, or catechins [3], especially in batch analysis, but also using flow injection analysis, for example by injecting antioxidants that can quench the peroxynitrite [9]. The microfluidic injection analysis presents different advantages such as the presence of laminar flow with no dilution effects, necessity of low volume of analyte (lower than 150 µL), miniaturization, the possibility of real-time continuous monitoring (process control), faster and more sensitive response, or the possibility of automated processes [10].

Electrochemistry uses usually low-cost instrumentation, has fast response time, and can be coupled with online analysis. Electrochemical methods are a better alternative than the usually used methods as they can assure direct, label-free, specific, real-time measurements. Different electrochemically active matrices are described in the literature, such as polymeric films (based on porphyrins, metal phthalocyanine, and/or conducting polymers) hybridized or not with graphene [7,11,12,13,14,15,16]. Only very few reports present the batch reduction (or oxidation at low potential) of peroxynitrite using a chemically modified electrode (presented in Table 1). Ligands based on extended π conjugated systems can create coordinative chemical bonds with different metals and act as good electrochemical mediators for different redox processes, even nowadays. Phthalocyanines (PCs) are part of this class, and due to different oxidation states of various metallic centers and high conductivity, they are a good platform for the detection of oxygen/nitrogen reactive species [11] or other molecules [17]. PCs are not toxic and have high thermal resistance and are quite stable at room temperature, assuring the stability of the biosensors in time. Except for the metallic centers, the ring-based redox processes may also influence the catalytical activity.

The sensitivity of chemically modified electrodes is greatly improved using a flow analysis system (FIA) compared to batch [18]. FIA is easier to use in comparison with batch; it increases the reproducibility and simplifies quantification. In addition, the optimization of a method is more rapid, and testing electrodes is more efficient [10]. Understanding the mechanism of reaction is very important, and FIA provides several advantages in electrochemistry that could be useful for this purpose. For example, by using microreactors, one can narrow the diffusion layers of the electrodes that could also “overlap”, which helps to optimize the reaction conditions and facilitates the determination of the mechanism. Channon et al. achieved pharmaceutical detection limits with an FIA electrochemical method for hydrazine detection [19]. They described that convective mass transport enhances the electrochemical signal by comparison with diffusive stationary experiments (batch). Recently, nanomolar levels were achieved for the detection of acetaminophen and codeine, using an FIA system combined with multiple pulse amperometry, and the analytes were also quantified in urine and human serum with excellent recoveries [20].

Herein, we describe the electrochemical reduction of peroxynitrite at 0.1 V, using a commercial cobalt (II) phthalocyanine complex (CoPc) and screen-printed carbon electrodes (SPCE). This electrochemical sensor is able to select between the most important interfering species of peroxynitrite (nitrite, nitrate, and hydrogen peroxide) and other molecules (e.g., ascorbic acid and dopamine), due to a specific, but simple design, combined with the advantages of a micro-fluidic system. 

In the last part, we show that our proposed method could be used to further study the decay kinetics of PON in the absence and presence of myoglobin. Our method is both a detection and quantification method and a further tool for kinetic studies, as the RSDs values between the classical static UV-Vis method and our method are low (less than 10%).

## 2. Materials and Methods

Sodium nitrite, hydrogen peroxide (30%), manganese dioxide (MnO_2_), myoglobin from equine skeletal muscle, sodium hydroxide (NaOH), sodium phosphate dibasic dihydrate (Na_2_HPO_4_ 2H_2_O), cobalt (II) phthalocyanine (CoPc), phthalocyanine (H_2_Pc), DMF (dimethylformamide), and TBATBF_4_ (tetrabutylammonium tetrafluoroborate 99%), hydrochloric acid (HCl), hydrogen peroxide 30% (H_2_O_2_), sodium nitrite (NaNO_2_), sodium nitrate (NaNO_3_), ascorbic acid, and dopamine were acquired from Sigma-Aldrich. Screen-printed carbon electrodes were acquired from DropSens, Spain. 

### 2.1. Peroxynitrite Synthesis

Peroxynitrite (PON) was synthesized following a slightly modified procedure [27]. Briefly, a solution of 0.7 M HCl + 0.6 M H_2_O_2_ was added over an ice-cooled stirring solution of 0.6 M NaNO_2_, and almost simultaneously, a solution of 3M NaOH was added over the mixture to quench the decomposition of peroxynitrite (a yellow solution). After several reaction minutes, a few grams of MnO_2_ (0.1 g/mL) were added to the mixture, to catalyze the decomposition of hydrogen peroxide. After the gas liberation was finished (approximatively 15 min), the MnO_2_ was filtered under vacuum, and the solution was divided into small aliquots (1 mL) and stored in the freezer (−20 °C).

### 2.2. Electrode Chemical Modification

The SPCE electrodes were modified by drop casting 2 μL of a (cobalt phthalocyanine) CoPc solution (1 mg/mL in DMF). The CoPc solution was prepared by dissolving CoPc in DMF (1 mg/mL), using ultrasonication over 1 h (power 100%, frequency 37 Hz). After the drop-cast, the electrodes were dried at 60 °C in the oven (for 15 min). Before different drop-casting steps, the electrodes were rinsed with DMF and dried with nitrogen. The process was repeated 3 times, without rinsing. The electrode was stabilized by cycling between −0.6 and 0.6 V (in PBS pH 12). Two reduction pre-treatments were proposed for the optimization of the SPCE/CoPc for PON detection: amperometry at −0.3 V for different time periods and chemical reduction with 25 mM sodium borohydride, during 20 min, followed by rinsing with deionized water.

### 2.3. Meat Extracts and Myoglobin Solutions

Manz meat (veal under the age of 2) was achieved from a local store. Yellow filtering paper (Filtrak n. 389), ROTI^®^Spin MINI-3 25 units CL12.1 (gel ultrafiltration, 1.5 Eppendorf tubes, for 3 kDa), and Sephadex G-25 in PD-10 Desalting Columns were used to remove the strong reducer (sodium borohydride) from the metmyoglobin (metMb) reduced system (redMb). The separation systems were bought from Sigma Aldrich. For oxymyoglobin (redMb) synthesis, a solution of 75 mM of sodium borohydride (NaBH_4_) in PBS pH 9 was added to a solution of 25 µM metMb.

The meat extraction was done according to the procedure from [28]. Briefly, 200 g of meat were cut in small pieces and blended with 100 mL of PBS pH 9. In addition, to the mixture, 400 mL of 0.1 M PBS (pH 9) were added, and the solution was stirred during 30 min, on an ice bath. After stirring, the mixtures were centrifuged 20 min, at 15 °C, at 9000 RPM, and the supernatant was centrifuged in the same conditions. Filtration on yellow filter paper was performed under vacuum and the pH was adjusted to 9, using sodium hydroxide. The desired pH values of the solutions were 12, 9, and 7.4, and the concentration of the phosphate was 100 mM. 

For PBS pH 9, Na_2_HPO_4_∙2H_2_O (0.1 M) and KCl (0.1 M) were dissolved in 500 mL of ultra-pure water. The solution of pH 12 was prepared in the same way, but NaOH was added: 450 mL solution was titrated with NaOH (approximately 35 mL of 1.4 M NaOH) until pH 12 and brought to 500 mL at the end. For PBS, pH 7.4 prepared tablets were used. As we designed the synthesis of PON to obtain high-concentration stock solutions and only added very small amounts of alkaline PON solutions to PBS pH 9 buffer, the pH 9 was practically constant [29].

### 2.4. Electrochemistry

A single-line flow system was coupled with potentiostat using a flow cell provided by DropSens, for the SPCE electrodes (DRP110). The Electrochemical Flow Injection Analysis system (FIA-EC) used is composed of a four-channel Peristaltic Pump—MINIPULS^®^ 3 (Gilson, Villiers-le-Bel, France), Injection Valve 77521 Rheodyne (with a 100 µL sample loop), Flow Cell from DropSens (model DRP-FLWCL), and Potentiostat (Autolab PGSTAT 101). A boxed connector for screen-printed electrodes from Dropsens was used to connect the SPCEs. Each measurement was performed in triplicate.

### 2.5. Determination of Apparent Rate Constants and Half-Lives

The kinetics of PON decay and the scavenging effect of myoglobin on peroxynitrite at pH 9 were assessed using the calculation of half-lives and apparent rate constants of peroxynitrite decay. The static method was used for the measurements of concentrations over time, as pH 9 offers the possibility of having slower decays and changes dynamics toward specific chemical decay reactions. The terms “peroxynitrite” and “PON” are widely accepted for a mixture of ONOO^−^ and ONOOH, depending on the pH. At pH 9, ONOO^−^ is assumed to be in excess over the protonated form ONOOH, based on the pKa of PON 6.8. The term “peroxynitrate” refers to a mixture of O_2_NOO^−^ and O_2_NOOH, depending on the pH. Trivial names: ONOO^−^, peroxynitrite; ONOOH, peroxynitrous acid; O_2_NOO^−^, peroxynitrate; O_2_NOOH, peroxynitric acid.

For the determination of the kinetic parameters, we took in consideration the previously proposed model by which the bimolecular decomposition of PON is dominant at pH 9, according to the following reaction [30,31]:

HOONO + ONOO^−^ −> 2 NO_2_^−^+ O_2_ + H^+^ + 2e^−^ (1)

According to this model, the rate law should follow pseudo first-order kinetics at pH 9, due to the excess of ONOO^−^. Our aim was to validate the FIA-EC method as a tool for the determination of rate order and kinetic parameters by comparing the results with the classical UV-Vis method. In this particular case, the first purpose was to establish by both UV-Vis and FIA methods if the reaction follows indeed pseudo first-order kinetics or second/higher-order kinetics at pH 9.

Two approaches were used for the confirmation of rate order and the calculation of apparent rate constants and half-lives: the first method (namely called from now on “Method A”) uses the plotting of all the integrated rate law data, according to the assumed rate order. Briefly, this was done as follows: the linearity (from the value of R^2^) of the graphs *ln(concentration)* (for (pseudo) first-order) or *1/concentration* (for second-order) vs. *time* and the correlation of the observed half-life (extrapolation from the graph t_1/2obs_) with the calculated half-life (t_1/2calc_). The half-lives for (pseudo) first-order and second-order reactions were calculated with the following formulas, respectively: ln2/k and 1/k∙C_0PON_. C_0PON_ is the initial concentration of PON and k is the (apparent) rate constant. For the linearity, we considered the R^2^ values. If R^2^ approaches 1, is significantly higher than the R^2^ of the other model, *and* there is good similarity between t_1/2calc_ and t_1/2obs_, the corresponding apparent order of the reaction is attributed to the detriment of the other apparent order. Each decay rate constant determination was plotted for a total of 180 s.

The second method (namely called from now on “Method B”) was the “half-life method” described by Ira Levine, in the “Physical Chemistry” book, chapter 16, “Reaction kinetics” [32]. This method can be applied when the rate law has the form r = k[A]^n^. Based on Equation (1) and considering the excess of ONOO^−^, this method would follow the equation r = k[ONOOH]^n^, therefore determining the rate order in ONOOH. According to this method adapted to our particular case, one first plots the *concentration* vs. *time* and then should be able to fit the equation with a single-exponential decay function if n = 1 (based on Equation (2), where parameter k (the apparent pseudo first-order rate constant in our case, k_obs_) is solved after compilation. Secondly, one performs the extrapolation of t_1/2_ for various concentrations and then plots the *logt*_1/2_ vs. *logC*_0_ [32]. The slope of the logarithmic graph (that should have a linear fit) will establish the order of the reaction in reactant A, *n*, where *n* = 1 − *slope*. The two methods, A and B, were compared at the end.

C_PON_ = C_0PON_ × e^−kt^ (2)

where C_PON_ is the PON concentration at a specific moment in time.

Both A and B kinetic methods were used for the UV-Vis method and for our proposed FIA-EC method. The molar extinction coefficient of 1670 M^−1^cm^−1^ was used to calculate the concentration of ONOO^−^ at 302 nm [33,34].

### 2.6. Surface Characterization

Fourier transform infrared (FTIR) spectra were recorded using a ThermoScientific FTIR instrument (Nicolet 8700, Pleasantville, NY, USA) equipped with a VariGATR accessory (Harrick Scientific Products, Inc., New York, NY, USA). KBr pellets were used as reference, and the powders were ground with KBr to create a solid pellet. For the ATR measurement technique, the Perkin Elmer GX FTIR spectrometer equipped with a Pike MIRacle having a 1.8 mm round diamond crystal was used. Diamond has an intrinsic absorption from approximately 2300 to 1800 cm^−1^, which limits is usefulness in this region. 

Micro-Raman spectroscopy measurements were performed on a Horiba Jobin Yvon LabRam HRMicro-Raman system combined with a 473 nm laser diode as an excitation source. Visible light was focused using a 100× objective. The scattered light was collected by the same objective in backscattering configuration, dispersed by an 1800 mm focal length monochromator, and detected using a CCD.

SEM images were obtained using an electron microscope FEI-QUANTA 200 equipped with wolfram filament (W), at the ICECHIM laboratory (Bucharest, Romania). The SEM images were taken at an accelerating voltage of 25–30 kV using a gaseous secondary electron detector (GSED). 

Absorption spectra were recorded using Ocean Optics UV-VIS-NIR spectrometer, in the 200–1100 nm range.

The Limit of Detection and the Limit of Quantification were calculated with the following formulas: LOD = 3 σ_b_/m, and LOQ = 10 σ_b_/m, in which σ_b_ represents the standard deviation of the background and m is the slope of the calibration graph. The sensitivity of the sensor was calculated from the slope of the calibration curve (plot *concentration* vs. *current*). 

## 3. Results and Discussion

Detecting reactive oxygen and nitrogen species is of great importance for many domains. Peroxynitrite, despite being a short half-life oxidative species, induces powerful oxidative stress effects on cells. Scavengers are important tools to eliminate this oxidative stress. Myoglobin is one of the scavengers [35], as it is an oxygen-binding protein from the heme group. Cobalt phthalocyanine was already used in the literature as a bio-mimetic material for biosensors [36]. It has a similar scavenging role when it comes to PON. As the metal cobalt center of the heme, similar to iron, has multiple oxidation states, the reduction of PON seems to be catalyzed by CoPc through redox reactions. We demonstrate here that the Co^2+^/Co^1+^ redox couple is more effective than the high potential electrochemical methods reported in the literature for the electrochemical detection of PON, as it offers better selectivity. Cyclic voltammetry as well as other techniques (linear sweep voltammetry or differential pulse voltammetry) were used to determine that this redox reaction of PON is apparently an irreversible process.

Before presenting and discussing the results, several other important factors regarding PON are to be mentioned, which are factors that are also important challenges to overcome in developing a selective and sensitive sensor for PON for meat extracts. (i) PON is most stable in cold alkaline solutions, without any metals and carbonyl compounds [30] and even so, it slowly decomposes, mainly, to nitrite. (ii) Below pH 12, the decomposition rate increases, and at pH 7, the half-life of PON is below 1 s. (iii) Acidic pH favors the decomposition to nitrate. (iv) Temperature, buffers, or several scavengers (e.g., myoglobin in meat as in Figure 1) influence the stability of PON [30,31]. Proteins tend to precipitate at pH values around 12, so such alkaline environment is not suitable for our purpose. As the decay of PON is slower at pH 9 than at neutral pH values, pH 9 is an optimal compromise for the detection of PON. At this pH value, the decay to nitrite is the predominant pathway, and it depends on peroxynitrite concentration. Due to these factors, we needed a fast response technique to help us distinguish between PON, nitrite, and nitrate, and to combat batch problems (mixing of the aliquots added for detection would increase the noise of the measurement, the concentration of the analyte will not be homogenous, etc.).

### 3.1. Batch Determination of Peroxynitrite Using SPCE/CoPc Electrodes

#### 3.1.1. Characterization of the Deposited CoPc Films on the SPCE

**Figure 2:**
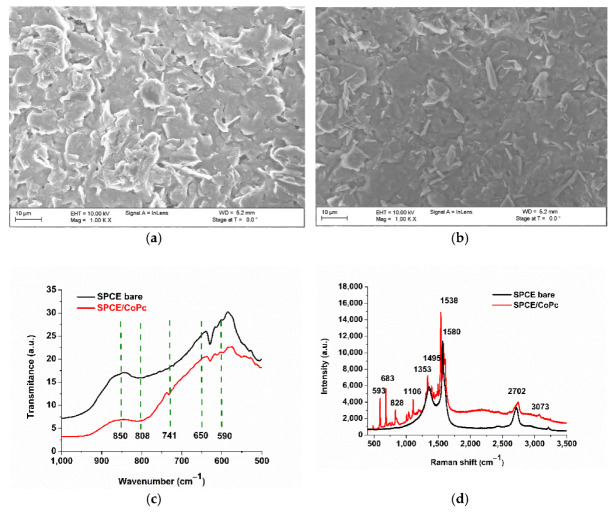
SEM characterization of the (**a**) unmodified SPCE and (**b**) SPCE/CoPc, (**c**) FTIR spectra of unmodified SPCE and modified SPCE with CoPc (SPCE/CoPc) [1], (**d**) Raman spectra of unmodified and CoPc modified SPCE.

SEM analysis of the morphology revealed that in accordance with the literature, the CoPc molecules tend to form random agglomerates [37] of various sizes (between 0.4 and 10 µm in length and a few hundred nm in thickness and width) depending on the surface used for the deposition (Figure 2a,b). The thin films were obtained by drop casting CoPc, due to π stacking (as the macrocycle has 18 delocalized π electrons), similar to other materials, such as graphene. This method is deposition is cost-effective and rapid.

The FTIR analysis (Figure 2c) proves the presence of CoPc on the surface of the electrode, especially due to the 741 cm^−1^ band in the fingerprint region, corresponding to phthalocyanine in plane vibrations [38]. Other vibrations belonging to the graphitic structure of SPCE are also observed: deformation of C-C out of aromatic plane (650 cm^−1^), vibrations of C-H in the aromatic plane (850 and 808 cm^−1^).

In the Raman spectrum of CoPc (Figure 2d), there are active modes of the symmetry A1g at 592 cm^−1^ (benzene radial), 834 cm^−1^, B1g at 684 cm^−1^ (macro breathing and benzene radial), 1542 cm^−1^ (C=N stretching mode), and B2g at 1498 cm^−1^ (pyrrole stretch) [39,40]. The HUMO–LUMO gap energy of CoPc is 1.9 eV.

**Figure 3:**
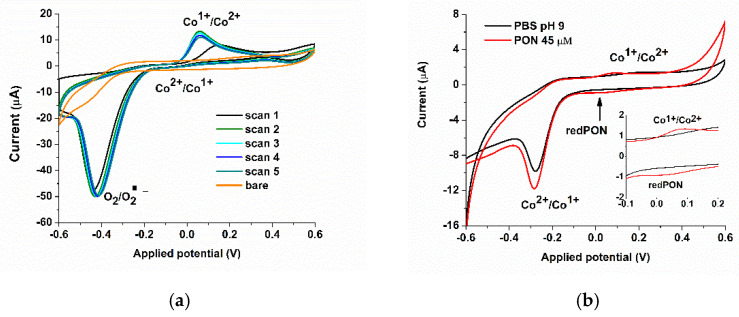
Cyclic Voltammetry (CV) of (**a**) the screen-printed carbon electrode (SPCE)/cobalt phthalocyanine (CoPc) electrode upon different scans, in PBS pH 12 [1]. The oxido-reduction processes are presented for the cobalt metallic center. (**b**) Cyclic voltammetry was registered using the SPCE/CoPc electrode in the absence (black) and in the presence (red) of 45 µM PON (scan rate 9 mV/s), PBS pH 9. Zoom in of the redox peaks for PON from the same plot.

After the morphological characterization of the surface of the electrode, we also used electrochemistry to extensively characterize the electrode. Cyclic voltammetry gave rise to two anodic and two cathodic peaks (corresponding to Co^3+^/Co^2+^ and Co^2+^/Co^1+^ redox processes), as previously reported [11]. In this paper, we targeted the exploitation of the Co^2+^/Co^1+^ redox couple (Figure 3a), due to the occurrence at more negative potentials (E_0_ ≈ 0.1 V), than the couple Co^3+^/Co^2+^ (E_0_ ≈ 0.65 V), and because, besides good sensitivity, there is the possibility to reach a remarkable selectivity for peroxynitrite. It can be observed that upon several scans (five cycles), the electrode reaches a steady state. Similar peaks were observed in the literature [41]. 

Cyclic voltammetry (CV), in the −0.6 to 0.6 V range, was used to determine the redox process of PON, using the SPCE/CoPc electrode, at pH 9 and pH 12. A higher pH value (12) presented enhanced anodic and cathodic peaks of the biosensor (Figure 3a) than a lower pH value (pH 9, Figure 3b, both in the presence and absence of PON), but high alkalinity interferes with the integrity of proteins (the final goal of our work) and may interfere with the integrity of the film, so it was not used further for analytical purposes, only for biosensor characterization. The redox process involving peroxynitrite at pH 9 takes place at around 0.1 V, with Ec = 0.047 and Ea = 0.072 (∆E = 25 mV = E_cathode_ − E_anode_), but there is a small current in the reduction, suggesting that the oxidation might be irreversible. The calculated formal potential (the redox standard potential, E^0^) is 0.06 V vs. Ag/AgCl pseudoreference electrode. Following the Nernst equation (Equation (3), where a_red_ represents the concentration of reduced species and a_ox_ represents the concentration of oxidized species, [32]) described below, we can conclude that two electrons are involved in the redox process (transferred in the cell reaction) at 25 °C. This conclusion is consistent with the nature of the peroxynitrite oxidant (usually described as a two-electron oxidant).

E_cell_= E_cathode_ − E_anode_ = E^0^ + 0.059/n ∙ lg a_red_/a_ox_ (3)

Furthermore, under another probable mechanism, an irreversible reduction takes place around −0.3 V, probably involving also the chemical oxidative reaction of peroxynitrite over the metallic center: cobalt being chemically oxidized by PON, which is electro-reduced, with a higher current, depending on the concentration of PON (Figure 3b).

**Figure 4:**
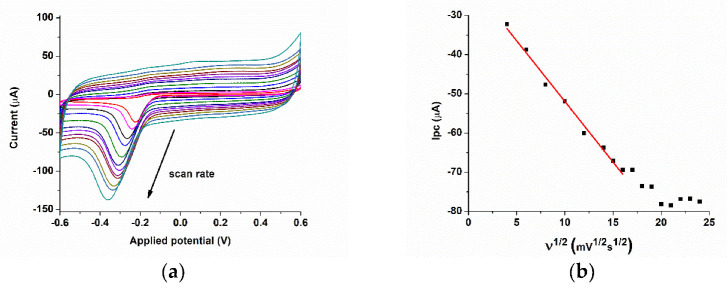
(**a**) Cyclic voltammetry of the SPCE/CoPc electrode for 50 µM PON, PBS pH 12. (**b**) The dependence of the cathodic current (around −0.3 V) with the square root of the scan rate.

If the anodic/cathodic current is proportional to the scan rate, the process is an adsorption-controlled process, and if the plot I vs. υ^1/2^ is linear, the redox processes are more likely diffusion-controlled ones [42]. For this purpose, cyclic voltammetry was used to determine the correlation between the cathodic current and the scan rate for scan rates in the range of 16–800 mV/s (Figure 4a). At lower scan rates, a linear correlation was obtained only for the square root of the scan rate, suggesting a diffusion-controlled process. Deviation from linearity usually means that other processes are involved, processes related to the surface, such as adsorption, ligand–species interaction, etc. At higher scan rates (above 256 mV/s), the correlation between Ipc vs υ^1/2^ is not linear anymore, suggesting more a mixture of surface and diffusion-controlled processes (Figure 4b).

**Figure 5:**
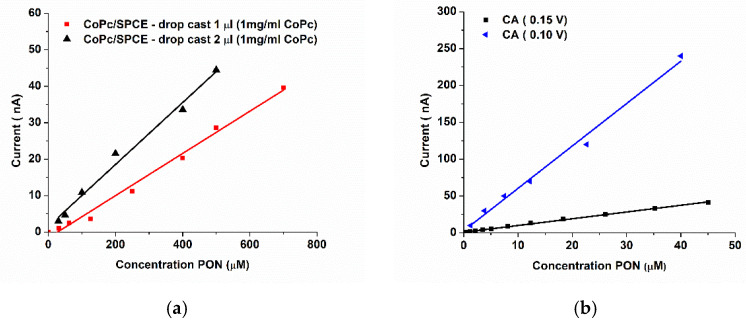
(**a**) Calibration curves for 1 µL and 2 µL drop-casted CoPc solution (1 mg/mL in DMF) on the SPCE (from DPV measurements) (**b**) Calibration curves from the amperometric response at +0.15 V (black) and +0.10 V (blue), using a GCE/CoPc electrode.

The SPCE is a three-electrode electrochemical system. The screen-printed area of the carbon-based working electrode (WE, black circle) of the SPCE is 0.126 cm^2^, which is surrounded by the Ag/AgCl pseudoreference electrode (silver) and counter/auxiliary electrode (CE, black). The chemical modification should not cover the CE and the reference electrode, but the surface coverage should be optimal. Starting from a solution of classical concentration for CoPc (1 mg/mL), we drop-casted 1 and 2 µL of the solution on the electrodes and calibrated them by DPV, from −0.4 to 0.3 V, step potential 5 mV, amplitude 25 mV, modulation time 50 ms, scan rate 10 mV/s (Figure 5a). The sensitivity for the 2 µL drop-casted CoPc was 0.083 nA µM^−1^, in comparison to 0.057 nA µM^−1^ for the 1 µL drop-casted CoPc. More than 2 µL is difficult to deposit without covering the other electrodes. These sensitivities are very low, but further optimization helps us improve them, especially using chronoamperometry at the optimized potential. The efficiency of different cobalt phthalocyanine layers was studied using Cyclic Voltammetry measurements. By drop-casting different layers of solution of CoPc, the best sensitivity for PON was achieved for three layers (drop-casting 2µL in three successive steps, that also included drying steps). The sensitivity for one layer of 2 µL CoPc was 3.3 nA µM^−1^, and for three layers of 2 µL CoPc, it was 8.5 nA µM^−1^, which is already one order and respectively two orders of magnitude improvements from the DPV method, but the LODs remained almost the same (5.45 µM and 5.14 µM, respectively). 

Chronoamperometry was used to study the preliminary potential for an improved quantitative detection of PON. Two different potentials were used, and both worked on the catalytical oxidation of PON with sensitivities of 8.75 nA µM^−1^ (0.10 V) and 0.91 nA µM^−1^ (0.15 V).

Based on the data described above, we propose a simplified mechanism of the catalytic process, involving two electrons as calculated from Nernst equation:

Co^II^PC + e^−^ → Co^I^PC (4)

(reduction)

2 Co^I^PC + ON^V^OO^−^ → intermediary complex → 2 Co^II^PC + N^III^O_2_^−^ + ½ O_2_ + 2e^−^ (5)

(oxidation/reduction)

In order to prove that the proposed mechanism involves the catalytic site of the metal phthalocyanine, we compared our electrochemical signals with the metal-free phthalocyanine (the exclusion of the catalytic site). Experiments were performed with H_2_Pc (dihydrogen phthalocyanine), which is the phthalocyanine ring without any metal coordinated to the nitrogen ligands. The H_2_Pc was not responsive to peroxynitrite, underlining the importance of the metallic center in this catalytic process (data not shown).

The surface coverage was calculated using the equation Equation (6):

Γ = Q/nFA(6)

where Γ is the coverage of CoPC immobilized upon the desired electrode surface (mol cm^−2^), Q is the charge taken from the integration of the oxidation wave resulting from the Co^1+^/^2+^ couple recorded in a pH 7.4 phosphate buffer solution (PBS) at slow scan rates, n is the number of electrons taking place in the electrochemical process, F is the Faraday constant, and A is the geometrical electrode area (without recourse to any surface roughness corrections, and it was calculated to be 0.126 cm^−2^, as the diameter of the WE is 4 mm) [43]. Using this calculation method, there was a surface coverage of 7.0558 × 10^−9^ mol cm^−2^ for the drop-casted CoPC SPEs, while the commercial (DRP 410, Drop Sense electrode) had a similar surface coverage (8.9643 × 10^−9^ mol cm^−2^). These kinds of commercial electrodes are recommended for the detection of hydrogen peroxide at low potentials (0.4 V).

#### 3.1.2. Batch Optimization of the CoPc-Modified Electrodes for PON Detection

**Figure 6:**
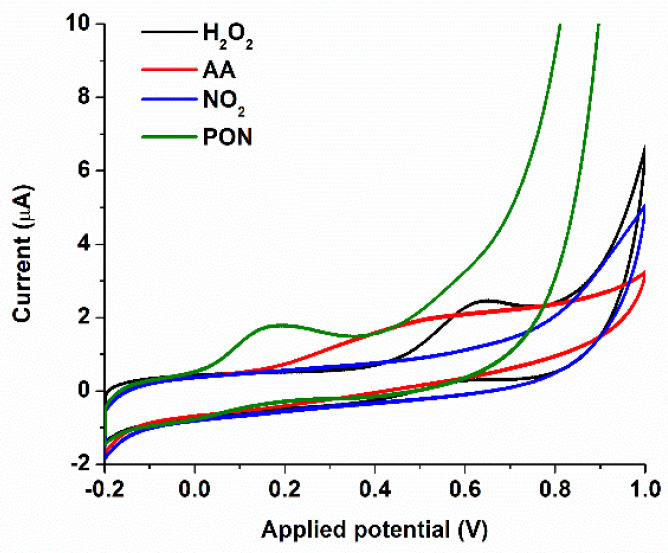
Evaluation of the interfering species using CV for 100 µM peroxynitrite (PON—green): 100 µM ascorbic acid (AA—red), 100 µM hydrogen peroxide (H_2_O_2_—black), 100 µM nitrite (NO_2_—blue), using a glassy carbon electrode (GCE)/CoPc electrode. Scan rate 100 mV/s, electrolyte: PBS pH 9 0.1M + 0.1 M KCl [1].

Bedoui et al. [44] mention that species such as ascorbic acid, nitrite and nitrate, uric acid, hydrogen peroxide, and others could interfere in blood or other biological samples with the signal that one may obtain for peroxynitrite. For this purpose, we studied a series of interfering species using Cyclic Voltammetry and a GCE-modified CoPc and compared the signal with the ones for PON at the same concentration, 100 µM (Figure 6). The biological concentrations of these interfering species are low, but we used the same concentration as for PON measurements, which usually is produced at a rate of up to 50–100 µM/min, but the steady-state reaction is in the nanomolar range, for hours [45]. Ascorbic acid gave rise to an oxidation peak around 0.4 V, hydrogen peroxide around 0.6 V, and PON was electro-catalyzed around 0.1 V (as already described above). Nitrate is not electrochemically active, and nitrite was not responsive within the chosen potential windows, meaning that using potentials around 0.1 V gives a very good selectivity toward PON.

**Figure 7:**
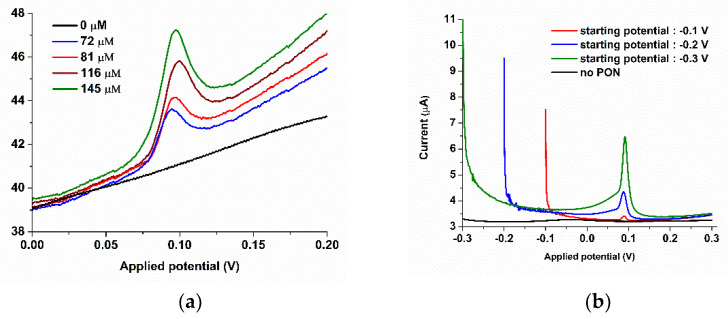
(**a**) LSV using SPCE/CoPc for the detection of PON, using droplets of analyte over the electrode, −0.4 to 0.3 V (electrolyte: PBS pH 9 0.1M + 0.1 M KCl), using different PON concentrations. (**b**) DPV of the SPCE/CoPc in the presence (red, blue, green) and absence (black) of 125 µM of PON, using different negative starting potentials (−0.1 V, −0.2 V, and −0.3 V).

More sensitive techniques than CV (Cyclic Voltammetry) such as LSV (Linear Sweep Voltammetry) and DPV (Differential Pulse Voltammetry) were used for further characterization of the electrodes. LSV voltammograms (Figure 7a) in the potential window of −0.4 to 0.3 V were used to observe the electro-oxidation of PON over the SPCE/CoPc electrode at different PON concentrations. The oxidation potential shifts from 0.075 V (for 72 µM PON) to higher potentials (around 0.1 V for 145 µM PON).

Differential Pulse Voltammetry (DPV) revealed that upon reduction of the CoPc films by starting the scans from lower potentials, the cumulative current (both cathodic and anodic) increases (Figure 7b). This suggests that the pre-treatment of the electrode could improve the response of the electrode for PON because of the reduction of Co^2+^ to Co^1+^. In addition, the shape of the DPV peak suggests that, besides diffusion, other processes occur (e.g., adsorption of product or reactant molecules on the surface of the electrode or even the coordination of the ONOO^−^ molecule to the metallic catalytic center). 

### 3.2. FIA Optimization of the SPCE/CoPc Electrodes for PON Detection

Initially, we did hypothesize that while the oxidation of Co^1+^ to Co^2+^ takes place, it can also be part of the redox process involving the reduction of peroxynitrite to nitrite. Starting from this hypothesis, an important issue was understanding how to overcome the apparent irreversible oxidation of PON from batch electrochemical analysis. The ability of flow injection analysis in understanding reaction mechanisms and complicated electrode kinetics [46] served as an important tool to select between the oxidation potential of peroxynitrite and of other species, such as hydrogen peroxide, and the electrocatalytic reduction potential of peroxynitrite was unraveled and exploited. 

**Figure 8:**
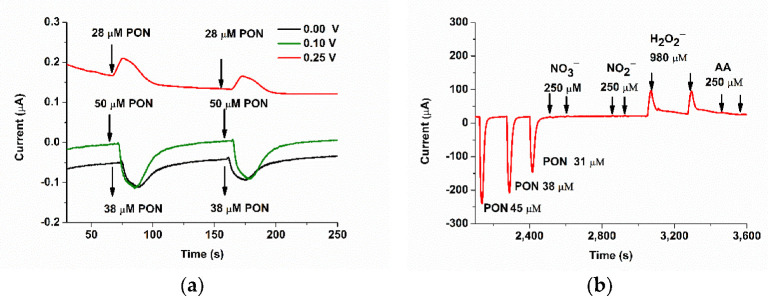
(**a**) Chronoamperometry (CA) spectra of the flow injection analysis (FIA) using different potentials: 0.00 V (black), 0.10 V (green), and 0.25 V (red) [1]. (**b**) Interfering species study using the FIA equipment and chronoamperometry at 0.1 V, in PBS pH 9: 250µM nitrate, 250 µM nitrite, hydrogen peroxide 980 µM and 250 µM ascorbic acid (AA), as compared to 31, 38, and 45 µM PON signals.

FIA coupled with chronoamperometry gave us the opportunity to develop a very selective sensor for PON, using the gathered information from the batch electrochemistry. First, we wanted to establish the optimal potential, so we used chronoamperometry and changed the applied potentials (0.00, 0.10, and 0.25 V) on a SPCE/CoPc electrode. This study helped us to determine that the reduction of PON occurs below 0.1 V, as opposed to the oxidation of PON that occurs above 0.1 V (Figure 8a). Due to the several other advantages already described, a single line flow injection system was employed for further experiments using the revealed reduction potential for further optimization of the PON sensor.

Determination of the optimum flow rate was done using injections of 200 µM of PON (PBS pH 12) and amperometry at 0.1 V. The curve *flow rate* vs. *current* was fitted with R^2^ = 0.9195 with a fourth-grade polynomial function. The optimal flow rate of 0.4 mL/min and a voltage of 0.1 V were used to further optimize the detection process, as the response time of this flow rate is fast, around 10 s. Interfering species were also evaluated using chronoamperometry at 20-fold higher concentration than PON for H_2_O_2_ and 5-fold higher concentration for the other species. As determined also by batch Cyclic Voltammetry, hydrogen peroxide gave rise to oxidation signals, but with very low sensitivity (Figure 8b) in contrast to PON, which gave rise to sensitive reduction signals. SPCE/CoPc electrodes are known to be used for the oxidation of hydrogen peroxide at 0.4 V.

As we have already shown for in batch electrochemistry (Figure 8b), the pre-treatment of electrodes with a reduction potential might be very important before the quantification of PON. We optimized the amount of time needed to reduce the CoPc film to obtain the best CA signal for PON. We used FIA amperometry for different time periods, 0, 20, 30, 60, 120, and 180 s and the reducing potential −0.3 V, which is the redox potential for Co^2+^/Co^1+^. We determined that applying a potential of −0.3 V for 60 s was the optimal procedure (data not shown).

**Figure 9:**
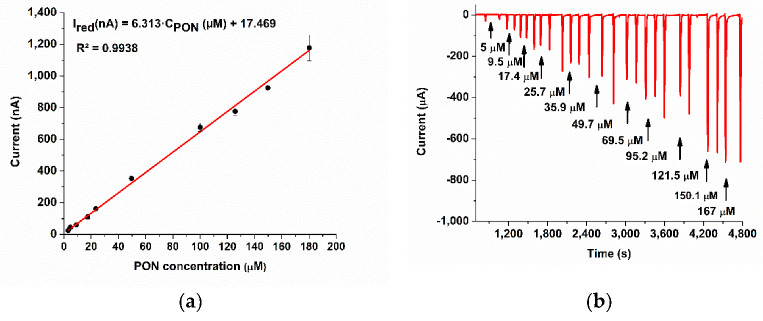
(**a**) Calibration curve of the SPCE/CoPc electrode for PON, PBS pH 9. (**b**) Chronoamperogram measured using the FIA system and the SPCE/CoPc electrode, E = 0.1 V, flow rate = 0.4 mL/min [1].

We have performed the calibration (Figure 9a) using the flow injection system, at 0.1 V with the chronoamperometric method (Figure 9b), and we obtained a sensitivity of 6.31 nA µM^−1^ (R^2^ = 0.9938), after the pre-treatment at −0.3 V for 60 s. The calculated LOQ = 2.41 µM, the calculated LOD = 0.72 µM, and the linear range is 3–180 µM. The reproducibility varied from 95% to 99% (50 µM PON) and the RSD for each calibration concentration (in triplicates) did not exceed 10%. The analytical parameters are very good if we consider several facts: (i) our unstable oxidative anion species are hard to detect, and (ii) screen-printed carbon electrodes are disposable electrodes.

By replacing the electrochemical polarization of the SPCE/CoPc electrode during the 60 s, at −0.3 V with the chemical oxidation of the CoPc film using sodium borohidrate (25 mM), for 20 min, the calibration was improved to Ired (nA) = 10.843·C_PON_ (µM) − 36.484 (R^2^ = 0.9925). The new LOD was equal to 0.42 µM, and the LOQ was 1.4 µM. 

The LODs of our method reached nanomolar level, the same level of PON under physiological conditions. Even though we did not study the interaction of PON in the absence or in the presence of myoglobin at physiological pH, our method has physiological relevance because it could be further used for this purpose by miniaturization of the electrodes with the same electrocatalytic bio-mimetic film. The micro-dimension of the surface-active area of the electrode offers enough sensitivity to study, for example, the formation of PON by cells (the cells being also in the micrometer range) involved in the oxidative burst (micromolar range of PON), in the redox signaling, or even in the steady state of PON (nanomolar range), as the literature suggests [47,48]. 

### 3.3. UV-Vis and Determination of Synthesized PON for Kinetic Studies

Molina et al. [30] describe the influence of buffer and pH over the stability of peroxynitrite solutions (“peroxynitrite” being the term widely accepted for peroxynitrite and peroxynitrate). The rate constants depend on pH, ionic strength, temperature, scavengers, and other parameters. Several mechanisms of PON decay have been already proposed in the literature. In acidic conditions, the isomerization of peroxynitrite occurs (mainly present as ONOOH, the form that decays rapidly to nitrate), independent of total peroxynitrite concentration. If pH is ≥7, nitrite is the main decomposition product of PON (the higher the pH value and concentration of PON, the higher the conversion yield to nitrite) [31]. As mentioned in Section 2.5, the Equation (1) describing the bimolecular decomposition of PON (as opposite to the mononuclear isomerization, as termed by IUPAC) is supposed to be predominant at pH = 9, especially when PON concentration is higher than 0.1 mM [32,49].

The reaction from Equation (1) was proposed to follow second-order or pseudo first-order kinetics, depending on pH [31].

Molina et al. [30] proposed that the decomposition of PON to nitrite has more intermediate steps than the one in Equation (1) (disproportionation reaction followed by the formation of intermediary/adduct species and then followed by decomposition to nitrite). The formation of the adduct is very rapid in the range of 10^4^ M^−1^ at alkaline pH. The last direct decomposition step to nitrite is much slower than the other elementary steps [31]. So, this could be the rate-determining step in these series of proposed reactions involved. In addition, the disproportionation of PON at pH 9 seems to favor the equilibrium toward ONOO^−^, as knowing the pKa of ONOOH (6.8), one can calculate the concentration of ONOOH at pH 9. For a concentration of 150 µM ONOO^−^ in PBS at pH 9, there are only 0.946 µM ONOOH, and for 50 µM ONOO^−^ in PBS pH 9, there are 0.3154 µM ONOOH. At pH 9, one can say that ONOOH concentration is insignificant (less than 1% of the ONOO^−^ concentration) if pKa 6.8 is taken in consideration. Nevertheless, the pKa of ONOOH depends on the ionic strength and pH, so these calculated values could be further refined [49].

If ONOOH is present in small amounts at pH 9, one can assume that the isomerization is insignificant (as the only form to isomerize is ONOOH) and PON decay should follow the pseudo first-order reaction at this pH.

Although the mechanism of PON decay is not fully understood, we took in consideration the generally agreed model depicted in Equation (1) for pH 9, as the aim was to determine the accuracy of the FIA-EC method compared to the classical UV-Vis one in determining the kinetic parameters. We checked both possibilities of reaction order for Equation (1), i.e., the pseudo first-order and the second-order.

We calculated the decay (apparent) rate constant and half-lives of PON using two methods: Method A involves the assumption that both (the (pseudo) first and second order) integrated rate laws could be possible. One finds the rate constant value from the slope of the two graphs: *ln(C_PON_)* vs. *time*, where t_1/2_ = ln2/k (for the (pseudo) first-order reaction) and *1/C_PON_* vs. *time,* where t_1/2_ = 1/(C_0PON_∙k) (for the second-order reaction). Using the slope of the most linear plot, one calculates the corresponding half-time. 

The second method, Method B, also named the “half-life method”, is based on the several half-time values from the *concentration* vs. *time* plot of the data, and it plots them after as *log(t_1/2_)* vs. *log(C_PON_*), where *n* = 1-slope (*n* being the (apparent) rate order). This method is more precise, as it is difficult to assess the linearity of a plot (as in Method A).

**Figure 10:**
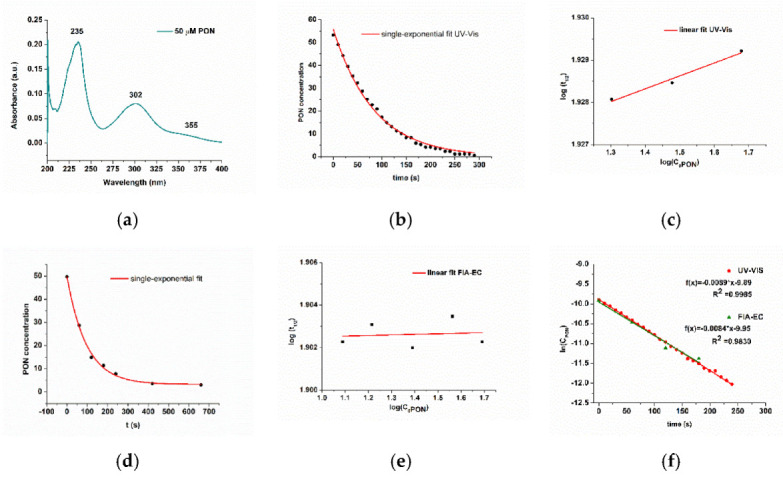
(**a**) UV-Vis spectra of 50 µM PON, pH 9, PBS 0.1 M. The illustration of the second method “half-lives method”: plot of *C_PON_* vs. *time*, fitted with a single-exponential equation (y = y_0_∙e^−kx^) for the data obtained using (**b**) the UV-Vis method and (**d**) our proposed FIA-EC method. Plot of *log(C_0PON_)* vs. *log*(*t*_1/2_) from (**c**) UV-Vis and from (**e**) FIA-EC, fitted linearly for the determination of apparent reaction order from the slope of the equation (Method B). The illustration of the first method (Method A): (**f**) plot of *ln(C_PON_)* vs. *time* according to pseudo first order, fitted linearly for both UV-Vis and our proposed FIA-EC method (linear correlation function, R^2^ linear correlation coefficient and apparent kinetic constant is determined from these plots, data presented in Table 2). All spectra represent the data for 50 µM PON, pH 9, PBS 0.1 M.

The idea was to compare the UV-Vis results with our proposed FIA-EC method using SPCE/CoPc electrodes. The UV-Vis spectrum of synthesized genuine PON is less complicated than other synthetic methods (such as using the nitric oxide and superoxide donor-based synthesis, SIN-1). The molar extinction coefficient of 1670 M^−1^ cm^−1^ can be used to calculate the concentration of genuine ONOO^−^ at 302 nm (Figure 10a) [33]. We evaluated the stability of our synthesized PON solution using UV-Vis spectrometry. As we have already described [1], because we performed our measurements at alkaline pH values, we had a significant amount of nitrite in the genuine PON solutions. The amount of nitrite is correlated with the absorbance at 355 nm. Calibration was performed at this wavelength using a Griess reagent-based protocol, (y = 0.0488·C_NO2_(µM) + 0.0076, R^2^ = 0.9971, data not shown), the amount of nitrite was also assessed to be 72 ± 5 mM for a 117 mM PON solution (improved PON synthesis), at pH 9, in PBS 0.1 M. This amount of nitrite is confirmed in the literature [24]. Nitrite reaches a plateau between pH 9 and 10 [50].

The kinetic plots at 302 nm (*concentration* vs. *time,* Figure 10b for UV-Vis data and Figure 10d for FIA-EC data) were fitted with a single exponential curve in Origin 8.5 software, as suggested in the literature [2,24,30,34,51]. The *log*(*t*_1/2_) vs. *log(C_PON_)* was plotted, and the slope was equal to *slope* = 1 − *n*, where *n* is the reaction order (Figure 10c,e). As it can be observed, Method B can be applied for both UV-Vis, as well as for the FIA-EC described in Section 3.2, and it will be further descried and compared with Method A (Figure 10f).

### 3.4. UV-Vis Determination of Different Forms of Myoglobin

**Figure 11:**
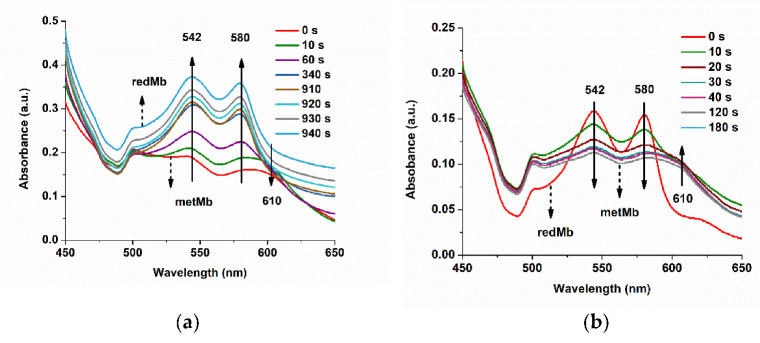
(**a**) UV-Vis spectra for different reaction times of 75 mM NaBH_4_ and 25 µM metMb (PBS pH 9). (**b**) UV-Vis spectra for different incubation times (0, 10, 20, 30, 40, 120, and 180 s) of 100 µM PON with 10 µM redMb (PBS pH 9).

In meat extracts, different oxidation forms of myoglobin are present, as we depicted in Figure 1. The conversion of reduced myoglobin (MbFe^2+^OH_2_ or MbFe^2+^O_2_) to metmyoglobin (MbFe^3+^(OH_2_)) can be followed using UV-Vis due to PON scavenging activity (Figure 11): the concentration of the redMb solutions can be verified by measuring the absorbance at 417, 542, and/or 580 nm (ε_417_ = 128 mM^−1^ cm^−1^, ε_542_ = 13.9 mM^−1^ cm^−1^, and ε_580_ = 14.4 mM^−1^ cm^−1^) [21], and the spectrum of metMb has a maximum of absorbance at 502 [ε_502_ = 10.2 mM^−1^cm^−1^] and 610 nm at pH 6.4, and the Soret band absorbance maximum is at 408 nM at pH 7.4 [52]. So, the scavenging effect could be identified using UV-Vis, but no quantification of PON decay can be done in a direct, rapid, sensitive, and selective manner.

The quantification of the concentration of redMb was realized with measurements at 580 nm. The concentration of the stock meat extract was determined to be 480 µM, which corresponds to ca. 20 mg of myoglobin for 1 g of meat. Moreover, if we use the absorption at 525 nm (representing the isosbestic point for the absorption in visible range for the 3 forms of myoglobin) and a molar extinction coefficient of 7.6 mM^−1^cm^−1^, we obtained a value of 485 µM of myoglobin for the same stock solution. A more complex method for determining the content of myoglobin was described by Krzywicki, and improved by Tang, in 2004 [53].

**Figure 12:**
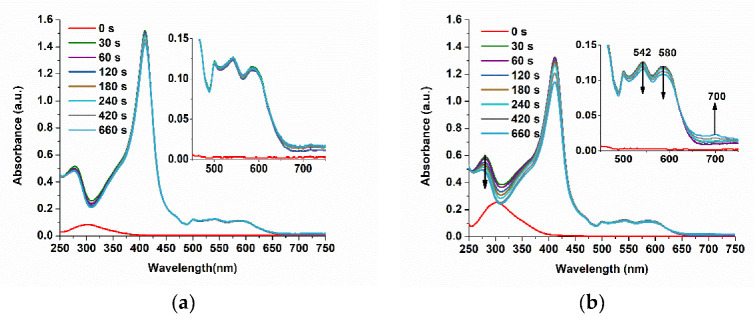
UV-Vis spectra of (**a**) 50 µM PON and (**b**) 150 µM PON incubated with 15 µM metMb, at different incubation periods.

The reactions of PON with myoglobin or meat were studied both with the electrochemical and spectrophotometric methods. Figure 12 describes the evolution of the Mb absorption peaks during Mb incubation with 50 and 150 µM PON. For PON 50 µM, almost no change was observed after 11 min of incubation, but the same incubation of metMb with 150 µM PON induced a more significant change, with decrease in absorbance at 542 and 580 nm, and the appearance of a band at 700 nm, corresponding to a qualitative evaluation of the catalytic reaction. The same incubation was studied with our FIA-EC method, also to obtain quantitative information regarding the catalyzed PON decay.

### 3.5. Studying the Reaction of Myoglobin with Peroxynitrite with FIA-EC

**Figure 13:**
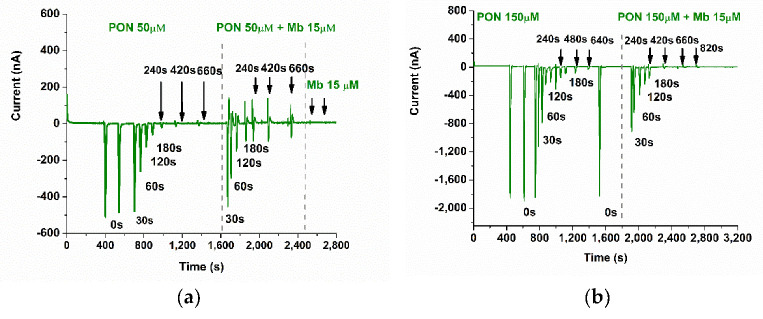
Chronoamperogram using the FIA-EC system for (**a**) 50 µM and (**b**) 150 µM PON, in the presence and in the absence of 15 µM metMb, at different incubation periods (0, 30, 60, 120, 240, 420, 660, 820 s). The decomposition of PON alone was studied at the same incubation periods to prove that the recovery of the current is due to the presence of PON in the metMb solution and not to the metMb itself (PBS pH 9, 0.1M, E = 0.1 V and flow rate 0.4 mL/min). The values obtained in these studies were further used for kinetic calculations.

The incubated solutions of 50 µM and 150 µM PON with 15 µM Mb were also investigated, in parallel, with our FIA-EC optimized method (Figure 13). It was expected that the kinetics between the two concentrations of PON to be different due to the ratio between PON and Mb, although the difference could be overcome by the kinetics of spontaneous PON decay independent of Mb, which presents opposite behavior. More precisely, increasing PON concentrations favor pseudo first-order reaction in relation to Mb and deviation to second-order reaction in the case of spontaneous PON decay. Moreover, approximatively at least eight equivalents of PON are necessary for the complete oxidation of myoglobin (depending on experimental conditions and pseudo first-order kinetics) [21]. The recovered current was converted into PON concentration with the calibration curve, and in the following sections, we discuss and compare the decay of PON using these data.

### 3.6. Studying the Reaction between Myoglobin from Meat Extracts and Peroxynitrite Using FIA-EC

**Figure 14:**
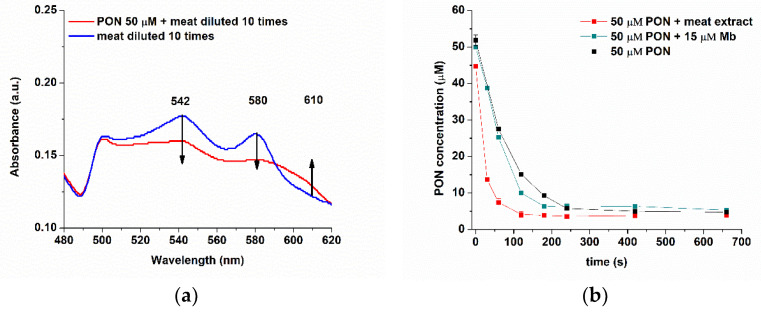
(**a**) UV-Vis spectra of meat diluted 10 times in PBS pH 9 in the presence of 50 µM PON and in absence, during 12 min. (**b**) The graphs of concentration (from FIA-EC) over time for 50 µM PON decay in the absence (black) or in the presence of 15 µM Mb (cyan) or meat extract (red).

Meat extracts were first diluted 10-fold and analyzed with UV-Vis to determine the quantity of myoglobin. Using the isosbestic point at 525 nm, as described above, we determined 15 µM of myoglobin (independent of the oxidation form, Figure 14a). The incubation of the meat extracts with PON was studied both with UV-Vis and FIA-EC. Using the optimized calibration curve of the electrochemical method, we developed (Ired (nA) = 10.843·C_PON_ (µM)–36.484, R^2^ = 0.9925), quantified PON during its incubation with both Mb 15 µM and meat extracts diluted 10 times in PBS pH 9, and compared it to a normal decomposition rate of PON, at pH 9, without any scavenger (Figure 14b). 

It can be clearly observed that the decomposition of PON takes place at a faster rate for the PON samples incubated with both Mb and meat extracts. 

Calculated apparent reaction orders, apparent rate constants, and half-lives determined using UV-Vis spectrophotometry and our FIA-EC method are presented in Table 2, Table 3, Table 4 and Table 5 and further discussed in the following two sections. All measurements and calculations were done for PBS pH 9, 0.1 M, at 25 °C.

#### 3.6.1. Estimation of the Apparent Rate Decay Orders of PON in the Absence and Presence of Myoglobin

Using Method A (Table 2), for 50 µM PON, the R^2^ values for the plot according to a first-order apparent kinetics (0.9985) were higher than for a second-order one (0.9246); as for 150 µM PON, the results were not conclusive (0.9872 for pseudo first order and 0.9877 for second order). Taking in consideration Equation (1) and the excess of ONOO^−^ comparing to ONOOH, these results from Method A are relevant and indicate pseudo first-order kinetics.

Using Method B, the apparent decay of PON is also a pseudo first-order reaction for both 50 µM and 150 µM (see Table 3), with calculated order values of 1.0030 and 1.0054 for the UV-Vis method, in comparison with 1.0000 and 1.0001 for the FIA-EC method. The differences between these two methods were less than 3% (acceptable error values). The fitting curves for both 50 µM PON (Chi-Sqr = 3.9770, R^2^ = 0.9865) and 150 µM PON Chi-Sqr = 1.9293, R^2^ = 0.9990) were proper, as tolerance criteria were satisfied.

Our results are in accordance with the literature, describing that especially concentrations above 100 µM present deviations from (pseudo) first-order reactions as second-order in total peroxynitrite concentration at pH 9 [30,31]. These deviations are most pronounced at pH 9 [50].

Method B helped us to assess numerically the reaction order out of our data acquired with the FIA-EC method for the interaction of 50 and 150 µM PON with 15 µM Mb, and the calculated reaction orders were 1.0222 and 1.0001, suggesting also a pseudo first-order kinetics in both cases. Deviations in apparent second order from the single-exponential curve were higher in the case of 50 µM PON + 15 µM Mb, as expected (meaning that the model could be adjusted, as pseudo first-order kinetics were not fulfilled for PON:Mb, Chi-Sqr = 15.7763, R^2^ = 0.9446). These deviations were smaller for 150 µM PON + 15 µM Mb (Chi-Sqr = 17.9047, R^2^ = 0.9941), as pseudo first-order kinetics were fulfilled: PON: Mb was 10:1.

Using both methods (A and B), the only studied interaction that gave clear second-order decay for PON was the meat diluted 10 times with 50 µM (R^2^ = 0.9020 vs. R^2^ = 0.9968 for (pseudo) first order and second order, respectively, using method A). The reaction order assessed with Method B is 1.94, which is very close to a second order. One explanation can be that in meat extracts, the interactions are more complicated/complex than with standard Mb, as other scavengers could be present, so an apparent second-order decay is easier to determine even using Method A (which is less precise than Method B). Method B will be used further on. Nevertheless, the fitting should be replaced with a more significant equation in this case (Chi-Sqr = 11.10, R^2^ = 0.9452), because this high deviation from a single-exponential equation proves once again that a second-order decay might be involved. A double-exponential equation could be helpful, as suggested in reference [54]. 

Nevertheless, the single-exponential fitting of pseudo first-order situations (C_PON_ = C_0PON_∙e−^kx^) determines *k_obs_*, which allows us to detect the (apparent) second-order rate constants (k_cat_), for a larger concentration range of scavengers or other reactants involved (especially the concentration of the catalyzer, myoglobin, or other) [24,25]. The k_cat_ obtained from a linear fit of *k_obs_* vs. *catalyst concentration* will refine our findings on second-order kinetics and improve the investigation of the scavenging effect of myoglobin over PON in meat extracts or other biological samples.

#### 3.6.2. Determination of Apparent Rate Constants and Half-Lives for the Decay of PON

Molina et al. [30] describe the observed half-life t_1/2_ = 9.4 ± 0.1 s for PBS 0.07 M, at pH 8, for PON 250 µM. We have determined that apparent k = 0.0086 s^−1^ with Method A and k = 0.0078 s^−1^ with Method B (the apparent first-order decomposition rate constant), for PBS 0.1 M, at pH 9, for a concentration of 150 µM (t_1/2_ = 252.12 ± 2.97). As it can be seen, in the chosen conditions, PON is more stable than in the conditions described by Molina et al., mainly because of more alkaline pH, more ionic strength in the buffer, and smaller concentrations of PON. Kissner et al. described this kind of behavior [31].

The decomposition of PON occurs faster at lower concentrations at pH 9, as it can be observed from the UV-Vis data in Table 3. The same conclusion can be drawn from our SPCE/CoPc developed method: PON 50 µM will decay faster (reaction rate of 0.426 × 10^−6^ s^−1^, using t_1/2_ = 81.33 s) than PON 150 µM (reaction rate of 0.412 × 10^−6^ s^−1^, using t_1/2_ = 252.12 s), where k = 0.693/t_1/2_ and r = k[C_PON_]. Reaction rates in the range of 10^−6^ for 50 µM PON at pH 9 are described by Kissner et al. [31], and this is in very good accordance with the data we obtained.

The myoglobin-mediated decay of PON is described as a second-order rate interaction. Most probably, the scavenging effect gives rise to ferrylmyoglobin (MbFe^4+^=O) with a rate constant of 4.6 ± 0.2 × 10^4^ M^−1^ s^−1^, at pH 8.3, in the absence of CO_2,_ that will further react with PON with a rate constant of 1.2 ± 0.2 × 10^4^ M^−1^ s^−1^ to form metMb [21]. The oxidation of redMb to metMb is not a stoichiometric reaction, as eight to 25 equivalents of PON are required for the oxidation reaction to be completed (depending on the absence or presence of CO_2_), as the natural decay of PON takes place at the same time. A 10-fold excess of PON is necessary for pseudo first-order conditions to be fulfilled. As the concentration of nitrite does not influence the interaction of myoglobin with peroxynitrite in a direct manner [21], our PON synthesized using the alkaline method is suitable for kinetic calculations (or otherwise saying, the decay of PON in the presence of myoglobin is a zero-order reaction in nitrite [2,21]). 

The metMb scavenges PON at a lower rate than redMb or even than ferrylMb. MetMb catalyzes the isomerization of PON to nitrate, at pH 7, at 20 °C, with a rate of 29,000 ± 100 M^−1^s^−1^, and an iron Fe^3+^ core of metMb is involved in this process. The k values decrease with increasing pH; thus, the decay rate is expected to be smaller for pH 9 [24]. So, scavenging PON with metMb is less effective at pH 8 (or above) than at biological pH, with a PON decay rate constant k of 2700 ± 30 M^−1^s^−1^, for pH 8, 0.1 M PBS, for 100 µM of PON, in the absence of CO_2_, at 20 °C [24]. In our case, at pH 9, PBS 0.1 M, 25 °C, the apparent k value of the reaction of 15 µM myoglobin and 50 µM PON was calculated to be 311.87 ± 7.96 M^−1^s^−1^ using Method B (where deviations from pseudo first order are second order). This value is lower than the value that Herold et al. obtained, which is probably due to the pH values and the temperature difference. As far as our knowledge goes, the kinetics of this exact decay conditions were not described in the literature before. Nevertheless, more PON or target molecule (myoglobin) concentrations are to be varied for refining better apparent second orders.

The scavenging was evaluated by comparing the decomposition rate of PON in the absence and in the presence of myoglobin (from both standard and meat extracts). The standard Mb decreased the half-life on PON from 81 to 65 s (for 50 µM) and from 250 to 88 s (for 150 µM), thus increasing the rate of decomposition in both cases. As in accordance with the UV-Vis spectra (Figure 12a) of metMb incubated with 50 µM of PON, the scavenging effect of 15 µM metMb with 50 µM PON is not very effective, and the calculated t_1/2_ values are in accordance with the UV-Vis data (with less 10% error values between the two methods). This is because the number of equivalents of PON (here around 3) were not sufficient to oxidize myoglobin. When we increase the number of equivalents to 10 (150 µM PON), the scavenging effect can be observed with both UV-Vis and FIA-EC. 

The scavenging effect of the myoglobin from meat was stronger than we initially thought (as we expected the concentration of Mb to be similar to the standard Mb), with a t_1/2_ of 19.77 ± 0.10 s. Even if we estimated the concentration of myoglobin to be around 15 µM in the extracted meat (diluted 10 times in PBS pH 9), when we compare the decay constants of PON, we can observe that the standard Mb has a lower apparent k constant (311.87 ± 7.96 M^−1^s^−1^) than the meat extract (891.76 ± 220.54 M^−1^s^−1^); thus, it has a higher half-life. Nevertheless, this may come from different oxidation states of the myoglobin in meat extract, and the presence of other possible scavengers in meat is not excluded. Other studies including varying concentrations of PON, myoglobin, and/or other catalysts from meat or other biological samples are still to be performed further.

## 4. Conclusions

Our label-free electrochemical method proposes a cobalt phthalocyanine deposition on the screen-printed carbon electrode (SPCE/CoPc) for the direct detection of peroxynitrite via electrocatalyzed reduction. This method is simple, sensitive, highly selective, rapid, and cost-effective. A simple flow injection system based on amperometric detection at potentials near +0.1 V was designed for PON determination, bringing the possibility for the automation and fast-response measurements. CA (chronoamperometry) was used to establish that changing the applied potential, the sensing mechanism of the electrode is changing/becomes easier to distinguish: the oxidation of PON occurs near 0.07 V and the reduction occurs near 0.05 V. At those low redox potentials (60 mV), the most important interfering species are less likely to appear, especially for the reduction potential. We optimized different parameters (flow rate, reduction potential, the quantity of deposited CoPc on the surface of the electrode, determined surface coverage etc.), and we suggested a mechanism that described the electro-reduction of PON, involving two electrons, based on the electrochemical characterization of the electrodes. We also determined that the electrochemical reactions taking place at the surface of the electrode are not simply diffusion-controlled ones, and other processes such as adsorption, ligand-based processes may take place. Interfering species were studied at a 5-fold concentration compared to PON, and only hydrogen peroxide responded with a very low sensitivity and under an electro-oxidation catalyzed mechanism, not influencing our PON detection.

Compared to the literature, this is one of the few articles based on the electro-catalyzed reduction of PON. Even though we obtained good sensitivity (with an LOD of 0.4 µM compared to 1.9 nM [14] and 1.0 nM [15], but higher than 5 µM [16]), we achieved a much better selectivity against all the important interfering species (ascorbic acid, hydrogen peroxide, nitrate, and nitrite) at biological concentrations. 

The optimized SPCE/CoPc calibration helped us monitor and quantify PON and the reaction between peroxynitrite and myoglobin. The literature presents the fact that the PON to redMb ratio must be 1:10 for efficient scavenging and that metMb is less efficient in scavenging PON (as the metal center is already oxidized) [24,52]. The scavenging of PON with myoglobin (in the form of metMb and redMb) decreases the amount of PON, and this decrease was measured/quantified with our SPCE/CoPc electrode. As the UV-Vis technique cannot be used to study the interaction with myoglobin (as the protein peak at 280 nm interferes with the PON peak at 302 nm), our FIA-EC method is an alternative technique that is able to study the scavenging effect of myoglobin from meat extracts toward peroxynitrite. The similarity of absorption spectra, especially in the UV zone, corresponding to redMb and meat extracts after incubation with PON, proving once again that PON is decomposed during the irreversible oxidation of redMb to metMb. 

We propose a simple method that has great impact on the PON sensor choice, as it can be used to quantify PON in complex media (such as meat samples) but also to study the kinetics of PON decay. We have also demonstrated that one can study the interaction of PON with different forms of myoglobin. Kinetic studies were also performed and correlated with the literature to study the scavenging effect. Meat extracts scavenged better PON, which was probably due to different forms of Mb and the possibility of another scavenger. The scavenging effect is a second-order decay rate (when PON is not in excess, when pseudo first orders apply), and the rate constants were determined.

Our study for the kinetics should be regarded more as an alternative possible method (“proof of concept”) for studying the interaction between PON and myoglobin using the FIA-EC method than a finalized kinetic study. The kinetic parameters obtained with both proposed kinetic methods (A and B) were in good correlation with each other. We can conclude that our FIA-EC method is precise enough for further studying the interaction of PON with scavengers. Evaluation of decay order for the interactions is impossible with the classical static UV-Vis method (that also has a low sensitivity toward PON, as an absorbance of 0.08 a.u. corresponds to 50 µM PON).

A further research direction is to use the already described hybrid materials (CoPc-graphene) to increase the solubility of the hybrid material in aqueous solvents and electron transfer at the surface of the electrode. The electroactive film could be also deposited on micro-electrodes to be used for the detection of PON in physiological conditions.

## Figures and Tables

**Table 1:** Literature study of the developed sensors used to detect PON via electroreduction.

Biosensor	Potential (V)	Sensitivity (nA mM^−1^)	LOD (nM)	pH	Ref.
Microelectrode Pt/Mn-pDPB (manganese-[poly-2,5-di-(2-thienyl)-1H-pyrrole)-1-(p-benzoicacid)]) coated with PEI (polyethyleneimine)	0.2	157.0	1.9	7.4	[14]
Nanoelectrode carbon fibers/manganese(III)-[2]paracyclophenylporphyrin	−0.35	1	50	-	[15]
Microelectrode Pt/MnTPAc (manganese tetraaminophthalocyanine)	−0.45	14.6	5000	10.2	[12]
Electrode SPCE/2,6-dihydroxynaphthalene	0.15	4.12	200	9–12	[16]
Electrode SPCE/cobalt phthalocyanine	0.1	10.84	400	9	this work

**Table 2:** Calculated pseudo first-order decay rates constants for PON, in PBS pH 9 (0.1M), at 25 °C, determined using classical UV-Vis method at 302 nm and the FIA-EC method, using the SPCE/CoPc (Method A).

Pseudo First-Order Decay Rates		k (s^−1^)			
	Method	SPCE/CoPc	R^2^	UV-Vis	R^2^
Samples					
PON 50 µM		0.0084 ± 0.0011	0.9830	0.0089 ± 0.0010	0.9985
PON 150 µM		0.0028 ± 0.0012	0.9975	0.0025 ± 0.0004	0.9877
Mb 15 µM + PON 50 µM		0.0134 ± 0.0010	0.9815	Not possible	-
Mb 15 µM + PON 150 µM		0.0086 ± 0.0020	0.9842	Not possible	-
Meat diluted 10 + PON 50 µM		0.0200 ± 0.0048	0.9020	Not possible	-

**Table 3:** The “half-life” method: Determined values of half-life for PON and calculation of rate orders. PBS pH 9 (0.1 M), at 25 °C, classical UV-Vis method at 302 nm and the FIA-EC method, using the SPCE/CoPc (Method B).

Half-Lives and Rate Orders	t_1/2_ (s)		Calculated Values for Rate Order *	
	SPCE/CoPc	UV-Vis	SPCE/CoPc	UV-Vis
PON 50 µM	81.33 ± 8.69	84.73 ± 3.53	1.0000 ± 0.0014	1.0030 ± 0.0016
PON 150 µM	252.12 ± 2.97	230.75 ± 5.13	1.0001 ± 0.0001	1.0054 ± 0.0009
Mb 15 µM + PON 50 µM	64.83 ± 1.64	Not possible	1.0004 ± 0.0008	Not possible
Mb 15 µM + PON 150 µM	88.12 ± 0.03	Not possible	1.0001 ± 0.0001	Not possible
Meat diluted 10 + PON 50 µM	19.77 ± 0.10	Not possible	1.9442 ± 0.0587	Not possible

* Rate order = n = 1- slope of the *log*(*C*_0_) vs. *log*(*t*_1/2_), the data were fitted with a single exponential function based on equation y = y_0_∙e^−kx^. Estimations of the rate orders were done in the case of values lower than 10^−4^.

**Table 4:** Observed first-order decay rates constants for PON, in PBS pH 9 (0.1M), at 25 °C, determined using the classical UV-Vis method at 302 nm and the FIA-EC method, using the SPCE/CoPc (Method B).

Apparent First-Order Rate Constants	k (s^−1^)	
	SPCE/CoPc	UV-Vis
PON 50 µM	0.00862 ± 0.0007	0.0080 ± 0.0018
PON 150 µM	0.00275 ± 0.0012	0.0030 ± 0.0004
Mb 15 µM + PON 50 µM	0.01690 ± 0.0010	Not possible
Mb 15 µM + PON 150 µM	0.00780 ± 0.0020	Not possible

**Table 5:** Calculated second-order decay rates constants for PON, in PBS pH 9 (0.1M), at 25 °C, determined using classical UV-Vis method at 302 nm and the FIA-EC method, using the SPCE/CoPc (Method B).

Apparent Second-Order Rate Constants	k (M^−1^ s^−1^)	
	SPCE/CoPc	UV-Vis
Mb 15 µM + PON 50 µM	311.87 ± 7.9600	Not possible
Meat diluted 10 + PON 50 µM	891.76 ± 220.54	Not possible
